# To the Editors of the Medical and Physical Journal

**Published:** 1809-05

**Authors:** 


### *To the Editors of the Medical and Physical Journal.*

GENTLEMEN,

IF the following Case will in any measure tend to illustrate the nature and treatment of disease, and is considered by you worthy of attention, the insertion of it in the next Nnmber of your useful miscellany will much oblige the writer,

Mr. L——d, a man about 30 years of age, applied to me a short time back, complaining of a violent pain in the abdominal region; there was no tension about the parietes, nor any increased action of the arterial system; the skin was cool, nor had he then any symptom that indicated inflammatory action; his bowels had been in a state of constipation for two days. I ordered him a saline purge to be taken directly, and an opiate draught at bed-time; next morning he sent me word that the opening medicine had not operated, and that he was in much more pain. He then took pills with the ext. col. comp. and calomel, with a purging mixture composed of the magnesia vitriol. &c. These in some degree removed the constipation, producing one or two motions. Towards evening he took a dose of an anodyne mixture, omni hora; the pain returned violently at intervals, so much so, that he could not be kept in bed. The abdomen was well fomented every ten minutes, and some lin. volat. rubbed in after each fomenting; he likewise took opii colat. gr. ß. omni hora, with a cooling saline mixture. This evening he took calomel gr. vj. in a pill, which was followed by a saline purge in the morning.—This plan appeared to give him some temporary relief, but next morning the pain returned with more violence than he had experienced before. The urine was scanty, and deposited a copious sediment; in short, he was now in so much pain, that he could not sit or lay in the same posture two minutes together. His friends being anxious, an eminent physician was consulted, who ordered him to take a draught three times a day with a saline mixture and infus sennæ, and between each of the draughts, four grains of the ext. pap. albi; a blister likewise was to be placed on the abdomen. He took the medicine once or twice without any relief. I had occasion to see him often in the day; and reasoning on the singularity of the case, and the little relief the remedies prescribed gave him, and collecting all the circumstances attending its its probable cause, a suggestion arose that it must be a spasmodic affection of the intestines induced by cold, as the patient, previous to the attack, had occasion to go into an ice house, and remain some little time. Upon this principle, considering that nothing was indicated but the free use of opium and aperients, he was ordered (while under the influence of violent pain) a mixture composed of t. opii ʒij. sy. papav. alb. ʒvj. aq. menth. ℥vſs. to take coch. iij. every half hour or hour, as the urgency of the pain required ; he likewise had a clyster thrown up, composed of ℥iv. ol. lini. and decoct. hordeat. I ought previously to have mentioned, that he had common clysters administered frequently before, to assist in keeping the bowels open, for through the whole of the attack, partial constipation attended. After he had taken the opiate medicine two or three times, the pain gradually left him. He is now in a state of convalescence, only wanting strength to bring him to his usual state of health. The only remark I shall make on the case is this, that the application of cold appears to me to have produced nearly the same effects as lead in the colica pictonum.

I shall leave the case, as stated, to the consideration of your readers, and if it should prove useful in any way, the writer's end will be fully answered.

OBSERVATOR.

*April* 11, 1809.

